# Inbuilt Mechanisms for Overcoming Functional Problems Inherent in Hepatic Microlobular Structure

**DOI:** 10.1155/2011/185845

**Published:** 2011-03-28

**Authors:** Robert D. Cohen, Christopher L. Brown, Carole Nickols, Pauline Levey, Barbara J. Boucher, Stephen E. Greenwald, Wen Wang

**Affiliations:** ^1^Centre for Diabetes, Bart's and The London School of Medicine and Dentistry, Blizard Institute for Cell and Molecular Sciences, Queen Mary University of London, Newark Street, London E1 2AT, UK; ^2^Department of Cellular Pathology, Bart's and The London NHS Trust, 80 Newark Street, London E1 2AT, UK; ^3^Medical Engineering Division, The School of Engineering and Materials Science, Queen Mary University of London, Mile End, London E1 4NS, UK

## Abstract

The spherical anatomy of human and rat liver lobules implies that more central cells have less time to carry out their function than more peripherally located cells because blood flows past them more rapidly. This problem could be overcome if more centrilobular cells could operate at higher temperatures than periportal cells. This study presents evidence for such a temperature gradient. Firstly, we use mathematical modelling to demonstrate that temperature increases towards the centre of the lobule. Secondly, we examine the distribution of a heat-generating protein and of a heat-sensitive protein across the rat and human liver lobules. Double-antibody staining of healthy liver from rat and human was used for visual scoring and for automated histomorphometric quantitation of the localisation of uncoupling protein-2 (known to generate heat) and of the transient receptor potential-v4 protein (known as a highly temperature-sensitive membrane protein). Both these proteins were found to be located predominantly in the centrilobular region of liver lobules. These findings support the suggestion that temperature gradients across the liver lobule may have evolved as a solution to the problem of reduced contact time between blood and cells at the centre as compared to the periphery of mammalian liver lobules.

## 1. Introduction

The mammalian liver is made up of approximately spherical lobules, approximately 1 mm in diameter [1]. Blood from the portal vein and hepatic artery flows centripetally from the periphery of the lobule between the cords of hepatocytes, exiting into a radical of the hepatic vein at the centre of each lobule. This structure implies that a hepatocyte in a more centrilobular zone has a larger volume of blood with which to interact per unit time than one more peripherally located, simple geometrical considerations showing that the most centrilobular tissue (*∼*75–85% down the radius towards the centre) has approximately 35 times the volume of blood to deal with per unit time than the most peripheral lobular tissue (0–10% down the radius). During evolution, mechanisms may have developed permitting centrilobular cells to function more rapidly than peripheral hepatocytes to compensate for their reduced contact time. 

 A rise in temperature from the peripheral to the central regions of liver lobules is one mechanism that would allow centrally located metabolic reaction rates to be increased. The use of purpose-built microthermocouples for the detection of temperature gradients has been attempted but was not feasible due to inevitable movement artefacts in the live animal and to possible errors from heat conduction along the probe. We have, therefore, sought alternative approaches to the investigation of this possibility. Firstly, the temperature increases to be expected as blood flow rate increases down liver lobules can be calculated. Secondly, we hypothesized that heat-generating proteins, such as uncoupling protein-2 (UCP-2) [2], might be present in greater amounts centrally as compared to peripherally, in the liver lobules and that proteins known to be highly temperature-sensitive, such as the transient receptor potential channels (TRPv4) [3, 4], might also be present in increased amounts centrally as compared to peripherally. Both of these mechanisms could contribute to a speed-up of centrilobular cell function which would assist in overcoming the reductions in metabolic efficiency resulting from reduced cell contact time as blood flows past centrilobular as compared to peripherally located hepatocytes. 

 In the present study, we have, therefore, modelled the temperature changes along the liver lobule that could be expected as a result of “wash down” of heat generated in exothermic peripheral reactions. We have also examined the location of two proteins referred to above along the lobular radius. The distribution of the transient receptor potential channel version 4 (TRPv4, a membrane channel constituent) along the liver lobule was examined as it is a temperature-sensitive protein with an unusually high *Q*_10_ (the increase of its functional rate for a temperature rise of 10°C) which is ×10–20-fold rather than the more usual ×2–5-fold [3, 4]. We also examined the distribution of the hepatic uncoupling protein-2, UCP-2 (which diverts energy generated in the electron transfer chain from ATP formation into heat production) along the liver lobule. Tight colocation of UCP-2 and TRPv4 to the centrilobular zone would support the hypothesis that evolutionary pressures have led to this situation because it achieves higher reaction rates in the centrilobular zone than in more peripheral zones. The present study was undertaken to examine the hypothesis that TRPv4 and UCP-2 are tightly co-located and are to be found predominantly in the centrilobular zone of the mammalian liver lobule, though not necessarily in the same cells, cell type, or bound at the molecular level.

## 2. Methodology

### 2.1. Mathematical Modelling

This was carried out using the dimensions of the human liver and liver lobules and the rates of hepatic blood flow and of hepatic heat production reported in the literature (see Results).

### 2.2. Liver Samples and Immunohistochemical Methodology

#### 2.2.1. Human Liver

3 *μ*m sections from formalin-fixed paraffin wax embedded (FFPE) blocks of liver from seven males aged between 21 and 63 years were used with the approval of the East London and City Regional Ethics Committee. On microscopy, the tissues used showed no autolytic changes or evidence of liver disease. A variable quantity of both lipofuscin and formalin pigments had to be accepted.

 An immunoperoxidase technique using Vector Laboratories ABC Kit (Ref: PK-7100) was employed on dewaxed sections following heat-mediated antigen retrieval, using Vector Antigen Unmasking Solution (Ref: H-3300 at pH 6.0). Sections for TRPv4 examination were held at 97°C in a water bath for 40 minutes. Sections for UCP-2 examination were microwave treated for 35 minutes. Sections were then incubated using Vector Laboratories Avidin/Biotin Blocking Kit (Ref: SP-2001) for 15 minutes, followed by peroxidase quenching with a 3% aqueous solution for 15 minutes. TRPv4 antibody was applied to sections at a dilution of 1 in 50 for 40 minutes. UCP2 antibody was applied to sections at a dilution 1 in 20 for 60 minutes. Both reactions were at room temperature. Visualisation of the target antigen was achieved using Vector(r) Nova RED(tm) SUBSTRATE KIT FOR PEROXIDASE (Ref: SK-4800) applied for 20 minutes in place of DAB (3.3′-diaminobenzidine chromogen solution) in order to circumvent difficulty distinguishing between the immune reaction and lipofuscin and formalin pigments. A light counterstain using haematoxylin (Gil's, made in house) for 5 seconds without differentiation proved optimal.

#### 2.2.2. Rat Liver

The animal work was done under the general institutional licence of the Department of Pathology for this type of animal study. Adult Glaxo-Wistar rats aged 6–9 months matched for age and sex and killed as control animals in an unrelated study had liver blocks fixed in formalin prior to processing to paraffin wax. 3 *μ*m sections from FFPE blocks of liver from 11 Wistar control rats aged between 6 and 9 months, matched for age and sex were used. The immunohistochemical method varied from that for human liver in a few details. Sections were treated with a peroxidase block of 3% hydrogen peroxide in methanol prior to antigen retrieval which for both antigens was by the use of microwave for 25 minutes in Vector Antigen Unmasking Solution. TRPv4 antibody was applied at a dilution of 1 in 20 and UCP-2 antibody at 1 in 40, both for 60 minutes at room temperature. Visualisation of the target antigen was by use of DAB chromogen (BioGenexHK 130–5 K) for 5 minutes followed by counterstaining with Gil's haematoxylin.

#### 2.2.3. Scoring

Zones for the manual assessment of staining were defined as periportal (a mantle of 2-3 cells), centrilobular (a mantle of 2-3 cells surrounding the hepatic venule), and mid-zonal (comprising 3-4 cells centred halfway down the lobular radius). Intensity scoring was done by a trained observer. In the visual scoring, approximately 12 (range 7–16) lobules were assessed for each liver. The scale used was 0–3, depending on whether the proportion of hepatocytes showing unequivocal staining was zero, <15%, 16–40%, or 41–100%. 

#### 2.2.4. Validation of Observer Performance for Rat Liver

The observer's performance was validated by comparing visual scoring with that obtained using histomorphometry for 1 lobule of each of 6 of the rat liver sections (three stained for UCP-2 and three for TRPv4) by an independent observer (the automated method cannot be used for human liver because of confounding by the presence of lipofuscin pigment). The images were captured from a digital camera (Zeiss Axiocam MRc) with an image size of 1388 × 1044 pixels, with an Axiocam MR frame grabber and processed by a custom-written macro running under a Zeiss KS400 semiquantitative image analysis system. One field per zone was examined at a magnification of ×40, giving a field area of 0.059 mm^2^. Immunostained areas were identified by colour (red, green, and blue pixel intensity ranges obtained by sampling the appropriate parts of a representative microscopic field after judging by eye the values required to discriminate these areas from background). These pixel values were then applied to all fields examined from each animal to produce thresholded binary images. The area of immunostained material was then calculated automatically from the binary image and expressed as a percentage of the total field area.

#### 2.2.5. Data Analysis

Data was examined using SPSS v.15. Data are expressed as means ± standard errors of the mean (s.e.m.) and differences were examined using Students *t*-test where appropriate. *P* values <.05 were considered significant. “*n*” values are for the numbers of animals or estimations, as stated in the text.

## 3. Results

### 3.1. Mathematical Modelling of Heat Transfer from the Periphery to the Centre of the Human Liver Lobule

For simplicity, we deal firstly with heat transfer in a hypothetical lobule in which no mechanisms such as those suggested above in relation to UCP-2 and TRPv4 had evolved. Assume that the lobules are on average spherical, and, in the first place, that heat generation within the lobule is uniform. Let *q* be the rate of heat production per unit volume of tissue (units J/(m^3^sec)), *s*: specific heat of tissue, that is, heat required to raise the temperature of a unit volume of tissue by 1°C (J/(m^3^ °C)), *k*: thermal conductivity (J/(m sec °C)). *T* and *u* are temperature (°C) and blood velocity (m/sec), respectively at a radial distance *r* from the centre of the spherical lobule. The general equation of heat transfer is

(1) $$\begin{array}{c} \nabla \cdot \left(-k\nabla T+usT\right)=q-s\frac{dT}{dt}, \end{array}$$

where ∇ denotes spatial gradient and *t* is time. For steady state situations considered in this analysis, *dT*/*dt* = 0. Assume that diffusive heat transfer is negligible compared with convective heat transfer by the blood flowing down the sinusoids. This is intuitively likely to be the case, because of the high blood flow through the liver. The diffusive term *k*∇*T* can thus be neglected, giving

(2) $$\begin{array}{c} \nabla \cdot \left(usT\right)=q. \end{array}$$

**Figure 1:**
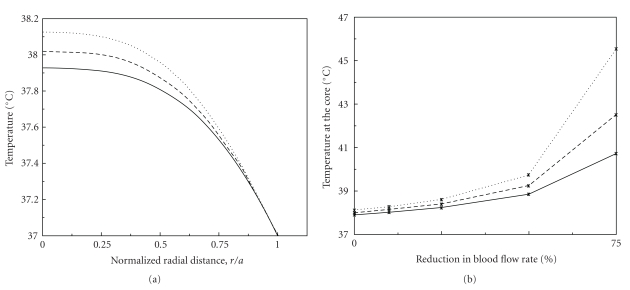
(a) Temperature distribution in the hepatic lobule at different radial distance from the centre, as predicted in the model, that is, in the absence of UCP-2. (b) Effects of reduced hepatic blood flow rate on temperature at the centre of the lobule. In both diagrams, the solid line (*Q*_10_ = 1) denotes the case when heat production rate is constant. The dashed line (*Q*_10_ = 3) and the dotted line (*Q*_10_ = 5) represent cases with a temperature-sensitive heat production rate.

Using Gauss' Theorem, this leads to

(3) $$\begin{array}{c} \iint_{\text{Surface}}usT\;dA={∭}_{\text{Volume}}q\;dV, \end{array}$$

that is, heat flux (*usT*) over the entire surface of a volume in the sphere equals the total heat production in that volume. Integration between *r* and the surface of the sphere, *a*, leads to (note that blood flow is in the −*r* direction)

(4) $$\begin{array}{c} s\left(r^{2}u_{r}T-a^{2}u_{a}T_{a}\right)=\frac{a^{3}-r^{3}}{3}q, \end{array}$$

where *T*_*a*_ and *u*_*a*_ are the temperature and average inflow blood velocity at the surface of the sphere. With volume conservation for the blood flow, that is, *r* ^2^ *u*_*r*_ = *a* ^2^ *u*_*a*_, the temperature distribution within the spherical lobule can be expressed as

(5) $$\begin{array}{c} T=T_{a}+\frac{V}{F}\frac{q}{s}\left[1-{\left(\frac{r}{a}\right)}^{3}\right], \end{array}$$

where *V* is the volume of a hepatic lobule, (4*πa* ^3^/3), and *F* is the blood volume flow rate entering each lobule, (4*πa* ^2^ *u*_*a*_). The temperature difference between the core (*r* = 0) and the surface (*r* = *a*) of the lobule is *Vq*/*Fs*. *T*_*a*_ may reasonably be assumed to be the temperature of the blood supply to the liver; in the case of the perfused liver, this is the temperature at which the perfusion system is kept—for example, 37°C. In the intact animal, this will be the core temperature. The specific heat of tissue, *s*, is taken as 3.57 × 10^6^ J/(m^3^_ _°C) based on a linear interpolation of the results of Haemmerich et al. [5]. In man, the volume of a hepatic lobule is approximately 0.179 mm^3^ (for *a* = 0.35 mm) and the average volume flow rate of blood into each lobule is approximately 0.1 mm^3^/min, based on a whole liver of 1.5 litre in volume receiving 0.83 litres of blood per minute [6], (assuming body surface area = 1.73 m^2^). The rate of hepatic heat production (*q* = 3.075 × 10^4^ J/(m^3^ sec)) employed in the present calculations is based on the mean value of 0.41 W per kg body weight, obtained in the anaesthetized dog by Baconnier et al. [6], by Leevy et al. [7], and Ersoz and Ersoz [8], assumes that liver accounts for 1/50th of the body weight (with a density of 1 kg/litre) and that hepatocytes occupy two-thirds of the volume of the liver (i.e., 0.41 × 50 × 3/2) and account for virtually all heat generation. The temperature distribution derived from this model could then be compared with that directly measured if this eventually becomes feasible.  Figure 1(a) shows the radial distribution of temperature given by (5), using the above values for the parameters; it indicates that the total theoretical temperature rise on moving from the periphery to the centre of the lobule is approximately 0.925°C.

 Now consider an alternative situation, also in the absence of UCP-2 effects, in which heat production *q *varies along the radius of the hepatic lobule such that *q*(*r*) is coupled linearly to the local temperature *T*(*r*) that is,

(6) $$\begin{array}{c} q\left(r\right)=q_{a}+\frac{T\left(r\right)-T_{a}}{10}\left(Q_{10}-1\right)q_{a} \end{array}$$

in which *q*_*a*_ is the heat generation rate at the surface of the hepatic lobule, where the temperature is *T*_*a*_, and *Q*_10_ is the increase in heat generation rate per 10°C rise in temperature. In spherical coordinates, the governing equation becomes

(7) $$\begin{array}{c} -\frac{1}{r^{2}}\frac{d}{dr}\left(r^{2}u_{r}sT\right)=\alpha +\beta T, \end{array}$$

where *α* = *q*_*a*_ − ((*Q*_10_ − 1)/10)*q*_*a*_ *T*_*a*_ and *β* = ((*Q*_10_ − 1)/10)*q*_*a*_. With volume conservation of blood flow, that is, *r* ^2^ *u*_*r*_ = *a* ^2^ *u*_*a*_, we can separate variables *r* and *T* in above equation:

(8) $$\begin{array}{c} \frac{1}{\alpha +\beta T}dT=-\frac{r^{2}}{a^{2}u_{a}s}dr. \end{array}$$

It can be solved, using the boundary condition at *r* = *a*, as

(9) $$\begin{array}{c} T\left(r\right)=T_{a}+\frac{1}{\gamma }\left(e^{\gamma (V/F)(q_{a}/s)(1-{\left(r/a\right)}^{3})}-1\right), \end{array}$$

where *γ* = (*Q*_10_ − 1)/10. The temperature difference between the centre and the surface of the lobule is (1/*γ*)(*e* ^*γ*(*V*/*F*)(*q*_*a*_/*s*)^ − 1).

In Figure 1(a), distribution of the temperature along the radius of the hepatic lobule at *Q*_10_ = 3 and 5 is also presented. Compared with the situation when heat production is uniform throughout the lobule, further increases in temperature in the central region of the lobule are seen when the heat production rate increases with the temperature. At *Q*_10_ = 5, temperature rise from the periphery to the centre of the lobule is approximately 1.12°C. In Figure 1(b), core temperature changes with reductions in hepatic blood flow (with exercise) are presented for situations where *Q*_10_ = 1, that is, there is no temperature boost due to UCP-2 and for proteins with a *Q*_10_ of 3 or of 5.

### 3.2. Immunostaining of Liver for UCP-2 and TRPv4

**Figure 2:**
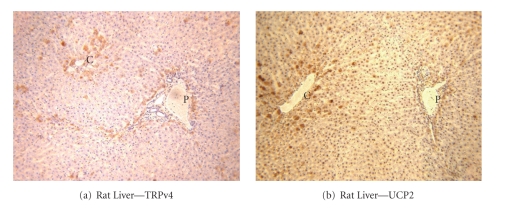
Rat liver: immunostaining showing co-localization of UCP-2 and TRPv4. Views across whole lobules with central and portal zones showing specific staining for TRPv4 in (a) and UCP-2 in (b), respectively. P indicates portal zones and C the centrilobular zones.

**Figure 3:**
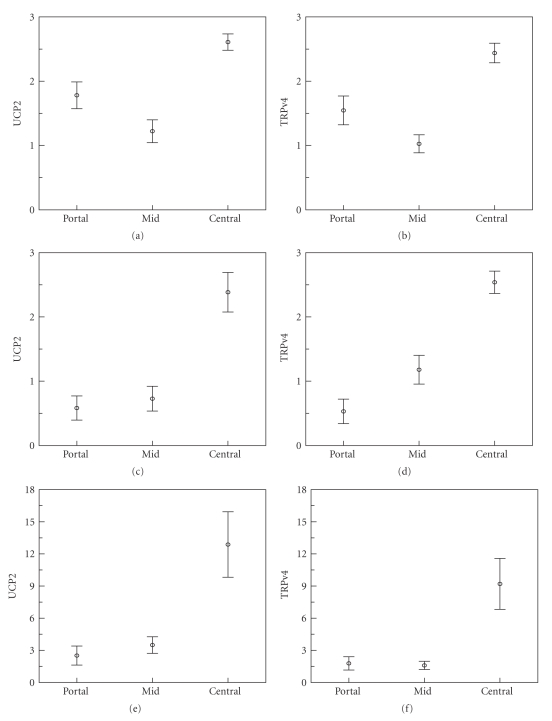
Mean UCP-2 and TRPv4 scores for human and rat liver. In rat liver ((a) and (b) manual scores; mean± s.e.m., *n* = 11) and in human liver ((c) and (d); ±s.e.m., *n* = 32). (e) and (f) show area fraction (%) UCP-2 and TRPv4 of immunostaining measured on automated histomorphometry in rat liver (mean and 95% CI, *n* = 6). Pairwise *t*-tests showed that the increases seen in central zones compared to either mid- or peri-portal zones on manual scoring in both rat and human liver were highly significant (*P* < .01), confirmed by significant reductions in % immunostaining in rat liver in mid and portal as compared to central zones on one-way ANOVA (*P* = .04 for UCP-2 and .001 for TRPv4).

Immunostaining of archival human and rat liver tissue for UCP-2 and TRPv4 (Figure 2) was cytoplasmic, appearing as coarse granules and blobs. Highest mean % immunostained areas were seen in the zone around the central vein in both rat and human livers (Figures 2 and 3). UCP-2 and TRPv4 were both heavily concentrated in the most centrilobular cells, both in human and rat liver (confirmed in the rat by automated histomorphometry, see below). Elsewhere, there was moderate staining, predominantly in the single layer of hepatocytes constituting the limiting plate of the portal tract, but very little in the mid-zones.

### 3.3. Automated Histomorphometry of Rat Liver

Automated histomorphometric measurement of the % area of immunostaining for both UCP-2 and TRPv4 in 6 rat livers, carried out to validate the visual scoring, confirmed the zonal differences found on manual scoring. (On one-way analysis of variance, automated histomorphometric data, in the rat liver, for mean % UCP-2 staining, was 9.63% centrally, 2.12% in mid zones and 1.46% in portal zones (*P* = .042) and for mean % TRPv4 was 7.095% centrally, 1.53% in mid zones and 1.63% in portal zones (*P* = .001).)

### 3.4. Correlation between Manual Staining Scores for UCP-2 and Trpv4

**Figure 4:**
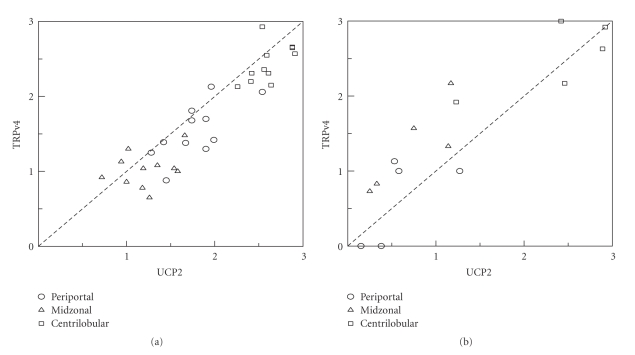
Correlation between manual staining scores for UCP-2 and TRPv4. (a) in rat and (b) in human livers. In rat liver Pearson correlation coefficient *r* = 0.86, *r* ^2^ = 0.74; in human liver *r* = 0.881, *r* ^2^ = 0.805 (data distribution is bivariately normal). ○: periportal; △: midzonal; □: centrilobular.

There was a strong correlation between manual staining scores for UCP-2 and TRPv4 at all sites taken together (see Figure 4); for example, in rat liver, the correlation coefficient between the two proteins for manual scoring was 0.895 (Spearman), suggesting that approximately 80% of the intralobular siting of UCP-2 and TRPv4 could be determined by the physiological requirement for TRPv4 (and other possible highly temperature sensitive proteins) to operate in a relatively warm environment. In human liver, the correlation coefficient, *r*, for manual scoring of TRPv4 and UCP-2 (Spearman) was 0.881, suggesting that *∼*77.6% of the intralobular siting of these proteins might be similarly determined.

## 4. Discussion

We have demonstrated, using mathematical modelling, that heat is washed down from peripheral to central parts of the liver lobule and have also shown that a heat-generating and an unusually heat-sensitive protein are co-located, predominantly in the centrilobular zones, in both rat and human livers. These data support the hypothesis that temperature gradients exist in liver lobules, with increases in centrilobular temperatures, that could compensate for the reduced contact time available for blood to interact with centrilobular cells, implicit in the spherical shape of liver lobules.

 These findings have several implications. Firstly, even in the absence of UCP-2, the centrilobular zone of the hepatic lobule would be expected to have a higher temperature than the more periportal regions of the lobule because of “wash-down” of peripherally generated heat, with an overall radial temperature gradient in the order of 0.9–1.2°K, depending on the intralobular distribution of heat production. Linear interpolation suggests that a protein such as TRPv4, whose particular function has a *Q*_10_ of 10–20 [2], would have that function increased by up to 2.4-fold (i.e., {1.2 × 20}/10) by a temperature increase of 1.2°K in centrilobular zones. Secondly, the convective “wash-down” of heat generated in periportal reactions is insufficient to produce the elevation of centrilobular temperature required to increase the rates of reaction (e.g., of glutamine synthesis, of detoxification by the cytochrome *P*_450_ system, or of glycogen formation via glucokinase [9, 10]) in the limited amount of centrilobular tissue whereas the additional heat generated by centrally located UCP-2 could do so. Thirdly, the centrilobular colocation of a protein whose function exhibits high temperature sensitivity (TRPv4) with the heat-generating protein UCP-2 suggests that this colocation could have evolved because of the functional benefits it provides to cells in the centrilobular zone. Fourthly, since there is a balance between the requirement for centrilobular ATP for biosynthetic, and other reactions, and the need to raise the centrilobular temperature for the reasons outlined above, the explanation for the additional, though less marked, colocation of UCP-2 and TRPv4 in the outer layer of peripheral cells is less obvious. It may be that the necessary rates of gluconeogenesis and ureogenesis, known to be maximal in the most peripheral zone in each lobule [11–13], are best achieved at the increased temperature provided by UCP-2 whilst high *Q*_10_ proteins such as TRPv4 might have some additional function, not yet identified, in this site. However, because of the particularly high requirements of gluconeogenesis and ureogenesis for ATP, the amount of periportal uncoupling is likely to be limited. 

 Further insights into lobular function may be gained by considering the changes during exercise. At the start of exercise, before body temperature has risen appreciably, the reduction in lobular blood flow [7, 8] may result in centrilobular warming; this may maintain centrilobular functions and thus limits glucose release into the general circulation because the activity of glucokinase (which is centrally located [10]) speeds up. At this stage, muscle glycogen is used to fuel muscular function. At a later stage, when muscle glycogen is exhausted, more glucose, derived from hepatic glycogen and from hepatic gluconeogenesis, is released and is available for fuelling muscle and brain. A limitation of the present study is that there is no ethical means of obtaining liver samples from absolutely normal human subjects. Samples were therefore obtained at autopsy from patients who had died from causes unlikely to result in liver pathology and where the liver had a normal histological appearance on routine staining. However, the similarity of the results in rats makes it likely that the centrilobular distribution of these two proteins is unrelated to cause of death in the human subjects and could well be a general mammalian phenomenon. A further limitation is the paucity of information on human hepatic heat production (*q*), and the resultant need to use data for *q* obtained in anaesthetized dogs for the mathematical modelling. 

 The theoretical calculation of purely convective temperature distribution (i.e., in the absence of boosting by UCP-2) along the radius of the lobule (5) depends on *a*, the lobular radius. In mammalian liver, lobules with mean radius greater than normal are found in at least three situations—in pregnancy [14], in regenerating lobules after partial hepatectomy [15], and in adult animals (rats) programmed by restriction of their mothers' protein intake during pregnancy and lactation [12]. This is of interest as (5) shows that the temperature difference between the surface of the lobule and the centre will increase with the radius of the lobule, likely to lead to further acceleration of centrilobular metabolism in larger lobules. This could be beneficial in the rat foetally programmed by maternal protein restriction where liver weight is not increased (so that there are fewer, but larger, lobules [12], and also during hepatic regeneration after partial hepatectomy [15]).

 Might the colocation of UCP-2 and TRPv4 occur by chance? Centrilobular staining for UCP-2 and TRPv4 together occupies approximately 0.1% of the lobular volume (excluding the hepatic venule), calculated from the automated area fraction data. The probability of centrilobular colocation of TRPv4 with UCP-2 occurring by chance is therefore only 10^−6^ (i.e., 10^−3^ × 10^−3^), further supporting the postulate that evolutionary pressures have resulted in the colocation of UCP-2 and TRPv4, a possibility supported by the presence of both these proteins in a single layer of hepatocytes at the lobular surface, whilst neither is detectable at any other site other than the centrilobular region. 

 If our interpretation of the role of centrilobular colocation of UCP-2 and TRPv4 is valid, how might this solution of the centrilobular problem have evolved? One possibility is that temperature-based compensatory mechanisms followed the physiological inevitability of the wash-down of heat generated in the periportal regions by exothermic reaction pathways; the resultant centrilobular warming being insufficient, the additional warming by UCP-2 proved beneficial. An alternative interpretation [16–20] of the function of UCP-2 in liver lobules is that it provides a mechanism which, by lowering the mitochondrial membrane potential, limits the production of reactive oxygen species and other free radicals (by-products of the respiratory electron transfer chain (ETC)), thereby reducing consequent cell damage by diverting energy released in the ETC from ATP formation to heat production. Although this could occur, this interpretation is not consistent with the radial distribution of UCP-2 we report since, if that were the case, one would expect UCP-2 to be concentrated predominantly in the periphery of the lobule since that is the main site of the highly ATP-dependent pathways of gluconeogenesis and ureogenesis and the site where reactive oxygen species generation as a by-product of the ETC is, therefore, likely to be at its greatest. However, the present study demonstrates the opposite situation, with the centrilobular zone showing much the highest UCP-2 concentration in both rat and the human. 

 Important effects could arise from the genetic polymorphisms of UCP-2 and its promoter region, reported in humans [21, 22]. Though the effects of these polymorphisms on UCP-2 function are unclear [2], loss-of-function polymorphisms, if not compensated for by increased UCP-2 protein activity, could contribute to glucose intolerance and Type 2 diabetes as a result of lower glucokinase-mediated centrilobular glucose uptake [4] due to decreased thermal stimulation. Similarly, loss-of function UCP-2 polymorphisms could also lead to increased risk of metabolic acidosis, due to a relative switch of ammonia detoxification from glutamine synthesis (centrilobular and proton-neutral) to ureogenesis (periportal and proton-generating) [23]. Gain-of-function polymorphisms could cause centrilobular cell damage as a result of repeated episodes of relative hyperthermia. Such effects might be especially likely during bouts of ethanol ingestion since alcohol dehydrogenase is known to be most active in the centrilobular zone [9]. Only a minority of heavy drinkers develop cirrhosis so that comparison of the frequency of the various UCP-2 polymorphisms in patients with cirrhosis with those without cirrhosis in a population of alcohol drinkers would be of interest. Finally, the current studies draw attention to the possible presence, and functional consequences, of temperature gradients at a microscopic level within the tissues, in this case in the liver, a field which has received little previous attention.
